# Supplementary material for: Generation of photonic entanglement in green fluorescent proteins
Source: Nat Commun. 2017 Dec 5;8:1934. doi: 10.1038/s41467-017-02027-9 (PMC5715022; doi:10.1038/s41467-017-02027-9)
Supplement: Supplementary file 1 — Supplementary Information [file 41467_2017_2027_MOESM1_ESM.pdf]

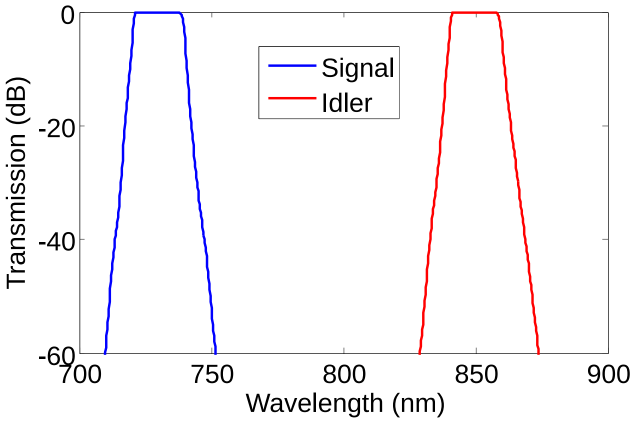

Supplementary Figure 1: (Color online) **The filter shapes of the signal and idler channels.** The filters are centered at 730 nm and 849 nm with FWHM of $\sim$ 20 nm, respectively.

Supplementary Note 1: Spectra and filtering

The spectra of $\omega_{\rm i}$ and $\omega_{\rm s}$ are obtained from the conservation of energy $2\omega_{\rm p} = \omega_{\rm i} + \omega_{\rm s}$ in the FWM process. In our experiment, the signal and idler spectra have flat top shape with a FWHM bandwidth of $\sim$ 20nm provided by the signal and idler filters as shown in Supplementary Figure 1.

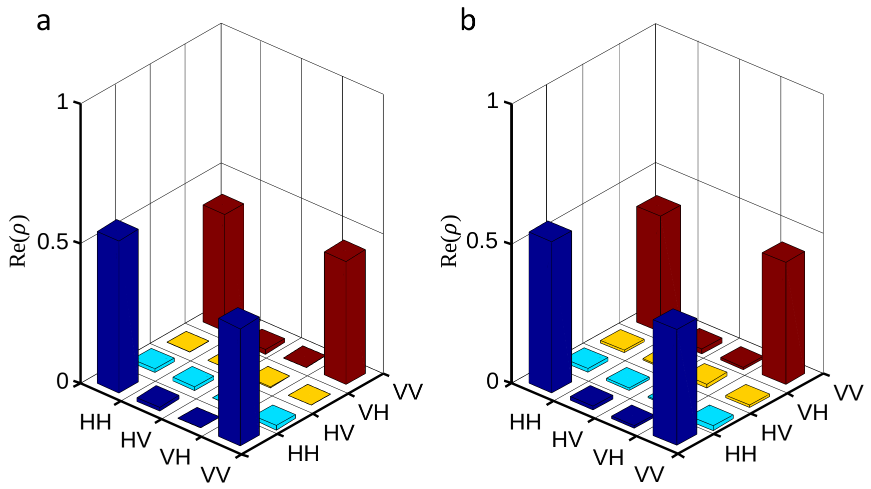

Supplementary Figure 2: (Color online) **Measured density matrices of entangled photonic states at 4-minutes and 6-minutes integration times.** (**a**) The reconstructed states in the horizontal-vertical basis for the integration time of 4 minutes. The fidelity is 0.92 ± 0.04. (**b**) The reconstructed states in the horizontal-vertical basis for the integration time of 6 minutes. The fidelity is 0.90 ± 0.03.

Supplementary Note 2: Quantum states of photon pairs

We can approximate the density matrix as $\rho'(p) = p|\Phi^+\rangle\langle\Phi^+| + (1-p)|H_\mathrm{s}H_\mathrm{i}\rangle\langle H_\mathrm{s}H_\mathrm{i}|$. $p = 0.89$ (0.88) for the integration time of 4 (6) minutes. We see that the probability p of different integration times are almost constant, which are consistent with the result from the 2-minutes QST measurement. We see that the fidelity of the state has decreased with integration time. This is due to a decrease in ρ_{HHVV} and ρ_{VVHH}. In theory, $\rho_{\mathrm{HHVV}} = \rho_{\mathrm{VVVV}}$, whereas in the experiment, ρ_{HHVV} is slightly less than ρ_{VVVV} with longer integration times. This is because the polarization interferometers may not be stable enough with longer integration times; the polarization interferometers may jitter during the experiment.
